# Effect of Dry Eye Disease on the Kinetics of Lacrimal Gland Dendritic Cells as Visualized by Intravital Multi-Photon Microscopy

**DOI:** 10.3389/fimmu.2020.01713

**Published:** 2020-08-12

**Authors:** Gustavo Ortiz, Cecilia Chao, Arsia Jamali, Yashar Seyed-Razavi, Brendan Kenyon, Deshea L. Harris, Driss Zoukhri, Pedram Hamrah

**Affiliations:** ^1^Center for Translational Ocular Immunology, Tufts Medical Center, Tufts University School of Medicine, Boston, MA, United States; ^2^Department of Ophthalmology, Tufts Medical Center, Tufts University School of Medicine, Boston, MA, United States; ^3^Program in Neuroscience, Graduate School of Biomedical Sciences, Tufts University, Boston, MA, United States; ^4^Department of Comprehensive Care, Tufts University School of Dental Medicine, Boston, MA, United States; ^5^Program in Immunology, Graduate School of Biomedical Sciences, Tufts University, Boston, MA, United States

**Keywords:** conventional dendritic cell, lacrimal gland, dry eye disease (DED), intravital multiphoton microscopy, kinetics

## Abstract

The lacrimal gland (LG) is the main source of the tear film aqueous layer and its dysfunction results in dry eye disease (DED), a chronic immune-mediated disorder of the ocular surface. The desiccating stress (DS) murine model that mimics human DED, results in LG dysfunction, immune cell infiltration, and consequently insufficient tear production. To date, the immune cell kinetics in DED are poorly understood. The purpose of this study was to develop a murine model of intravital multi-photon microscopy (IV-MPM) for the LG, and to investigate the migratory kinetics and 3D morphological properties of conventional dendritic cells (cDCs), the professional antigen presenting cells of the ocular surface, in DED. Mice were placed in a controlled environmental chamber with low humidity and increased airflow rate for 2 and 4 weeks to induce DED, while control naïve transgenic mice were housed under standard conditions. DED mice had significantly decreased tear secretion and increased fluorescein staining (*p* < 0.01) compared to naïve controls. Histological analysis of the LG exhibited infiltrating mononuclear and polymorphonuclear cells (*p* < 0.05), as well as increased LG swelling (*p* < 0.001) in DED mice compared to controls. Immunofluorescence staining revealed increased density of cDCs in DED mice (*p* < 0.001). IV-MPM of the LG demonstrated increased density of cDCs in the LGs of DED mice, compared with controls (*p* < 0.001). cDCs were more spherical in DED at both time points compared to controls (*p* < 0.001); however, differences in surface area were found at 2 weeks in DED compared with naïve controls (*p* < 0.001). Similarly, 3D cell volume was significantly lower at 2 weeks in DED vs. the naïve controls (*p* < 0.001). 3D instantaneous velocity and mean track speed were significantly higher in DED compared to naïve mice (*p* < 0.001). Finally, the meandering index, an index for directionality, was significant increased at 4 weeks after DED compared with controls and 2 weeks of DED (*p* < 0.001). Our IV-MPM study sheds light into the 3D morphological alterations and cDC kinetics in the LG during DED. While in naïve LGs, cDCs exhibit a more dendritic morphology and are less motile, they became more spherical with enhanced motility during DED. This study shows that IV-MPM represents a robust tool to study immune cell trafficking and kinetics in the LG, which might elucidate cellular alterations in immunological diseases, such as DED.

## Introduction

Dry eye disease (DED) is a significant public health concern affecting ~16 million adults in the United States alone (1, 2). DED is defined as “a multifactorial disease of the ocular surface characterized by a loss of homeostasis of the tear film, and accompanied by ocular symptoms, in which tear instability and hyperosmolarity, ocular surface inflammation and damage, and neurosensory abnormalities play an etiological role” (3). The lacrimal gland is the main source of the aqueous layer of the tear film, which is crucial to protect the cornea and conjunctiva from desiccation, infection and inflammation, thus preserving corneal transparency (4, 5).

Tear secretion is finely regulated by the lacrimal functional unit (LFU). The LFU is defined as the conjunction of lacrimal glands (main and accessory), the ocular surface and the communicating innervation (6). Importantly, this innervation is responsible for the maintenance of the tear film through baseline and reflex tearing. Like any neural reflex, this can be broken into an afferent and efferent arm. The sensory nerves within the cornea represent the afferent arm, detecting changes in the tear film osmolarity or mechanical stimuli, necessitating lacrimation. These signals are relayed to the LG through the parasympathetic nerves via the efferent arm, resulting in lacrimation. Thus, the homeostasis of the ocular surface and tear film stability can be compromised if any component of the LFU is damaged or dysfunctional, predisposing development of DED (7). Desiccating stress (DS) induced by housing animals in a low humidity controlled environment leads to recruitment of immune cells and enlarged secretory vesicles in the LG, suggesting that increased DS at the ocular surface may induce inflammation within the LG (8). Recently, a study showed that DED induced by DS affects sensory nerve density, morphology and function within the cornea (9). This study revealed that DED reduces the sensitivity of corneal nerves, suggesting that the afferent arm of the LFU may become dysfunctional. Furthermore, previous studies have shown that inflammation and neuropathic pain are common sequelae following spinal cord injury (10). Conversely, intact nerves can control inflammation within peripheral tissues by signaling to immune cells, preventing excessive damage to host tissues (11). Thus, there is a precedent for the notion that nerve dysfunction or loss of homeostatic signaling could lead to inflammation. We therefore hypothesize that compromised corneal sensory nerves on the ocular surface (afferent pathways of the LFU) may, in addition to decreased tear secretion, directly lead to LG inflammation.

Conventional dendritic cells (cDCs) are professional antigen presenting cells (APCs), responsible for the sensing of foreign antigens. cDCs are able to engulf, process, and present antigens from peripheral tissues to T cells within the draining lymph nodes. As such, cDCs link the innate and adaptive immune responses, and are critically involved in the initiation of immune responses (12). This holds true in the case of immune-mediated diseases as well, since cDCs have been shown to play a central role in the pathogenesis of DED (13). Contrary to long-held notions of corneal immune privilege, the cornea is not devoid of immune cells, and resident populations of cDCs have been identified in both the cornea (14, 15) and LG (16). DS has been shown to result in increased density of corneal cDCs (17) and decreased density of corneal nerve fibers (9). More recently, our group and others have shown that there is an inverse correlation between cDC and nerve density in the cornea (18–20). Moreover, our group has shown that greater corneal cDC motility was found during thermal cautery-induced acute corneal inflammation (21). However, the impact of DS-induced DED on cDC motility and kinetic properties in the LG, has not been investigated to date.

Intravital multiphoton microscopy (IV-MPM) enables studying the kinetics and 3-dimentional (3D) morphology of immune cells and cell-to-cell interaction *in vivo* over time (22, 23). By providing second harmonic generation, it also allows collagen delineation in the tissue (24). Thus, it has been widely used to study immune cells behaviors within solid tissues, including lymph nodes (25–27), bone marrow (28), cornea (21), skin (29), and the gastrointestinal tract (30). However, to date, IV-MPM has not been used to examine immune cell populations in the LG. Therefore, the purpose of this study was to first develop a novel IV-MPM model with proper regulation of temperature and tissue stability to study immune cell kinetics of the lacrimal glands, second to assess the 3D morphology of cDCs, and third to study cell kinetics of cDCs during DED.

## Materials and Methods

### Mice

Thy1-YFP mice (B6.Cg-Tg [Thy1-YFP]16Jrs/J) were obtained from the Jackson Laboratory (Bar Harbor, ME) as heterozygous and bred to homozygous with repeated matings between male and female mice with high copies of the transgenes for YFP for Thy1-YFP. This was required in order to obtain mice with higher fluorescence for IV-MPM. CD11c-EYFP mice were a generous gift from Dr. Michel C. Nussenzweig from Rockefeller University (27). Thy-1 x CD11c-EYFP mice were generated by crossing homozygous Thy1-YFP with homozygous CD11c-EYFP repeatedly until the both the nerves and DCs were co-localized with YFP in the cornea. Primer sets used for qPCR for genotyping: Thy1-YFP forward 5′- GCCCTGGCCCACCCTCGTGACCACCTTCG-3′ and reverse 5′- CCTGATGCCGTTCTTCTGCTTGTCGGGCA-3′, and CD11c-EYFP forward 5′- TGCTGGTTGTTGTGCTGTCTCATC-3′ and reverse 5′- GGG GGT GTT CTG CTG GTA GTG GTC-3′.

Thy1^YFP^ mice express YFP under the control of regulatory elements of the Thy1 gene, and thus label neuronal populations, primarily sensory and motor neurons. The CD11c^YFP^ mice carry EYFP transgene under the control of the CD11c promoter (27). Thus, our CD11c^YFP^×Thy1^YFP^ mice allow visualization of both CD11c^+^ cDCs and Thy1^+^ neurons in the same animals. C57BL/6N wild-type (WT) female mice were obtained from Charles River Laboratories, Inc. (Wilmington, MA). Because female gender is a risk factor of DED and female C57BL/6N mice develop greater corneal barrier disruption than age-matched males (31), in this study only 6- to 8-week old female mice were used in all experiments. Mice were housed at Tufts Department of Lab Animal Medicine and were treated in accordance with the Association of Research and Vision in Ophthalmology (ARVO) statement for the Use of Animals in Ophthalmology and Vision Research. All experiments were performed after the review and approval from the Institutional Animal Care and Use Committee (IACUC number B2018-47) at Tufts University and Tufts Medical Center, Boston, MA.

### Acute HSV-1 Keratitis

The herpes simplex virus (HSV)-1 McKrae strain (kindly provided by Dr. Homayon Ghiasi, Cedars-Sinai Medical Center, Los Angeles, CA), a stromal disease-causing, neurovirulent HSV-1 strain was used for corneal inoculation. HSV-1 was propagated in Vero cell cultures (American Type Culture Collection, Manassas, VA). Briefly, Vero cells were grown to confluence in T150 cm^3^ culture flasks and infected with 2 × 10^6^ plaque forming unit (PFU) of virus stock in 1.5 mL and tilted every 10 min for 1 h. Then, growth media (DMEM plus 5% FBS, both Corning Inc., Corning, NY) was added, and cells cultured for 3 days at 37°C. Afterwards, the maximum viral cytopathic effect was expected, and the infected cells were extracted after 2–3 cycles of cell lysis by using the GentleMACS dissociator (Miltenyi Biotec Inc, San Diego, CA). Then, the lysate was clarified by centrifuging for 10 min at 3,500 rpm at 4°C, followed by a spin down at 17,000 rpm for 30 min at 4°C. The virus pellet was re-suspended, aliquoted and stored at −80°C. Virus titers were determined by standard plaque assay after the infection of Vero cells as previously described (32).

Mice were anesthetized with a mixture of ketamine (120 mg/kg) and xylazine (20 mg/kg) and injected intraperitoneally. A drop of proparacaine hydrochloride (Akorn, Lake forest, IL) was applied for local anesthesia to the eyes before scarifying the cornea. One cornea (previously anesthetized with one drop of 0.5% proparacaine) per mouse was scarified in a 5 × 5 grid-like pattern along the cornea with a 30-gauge needle. Afterward, 3–5 μL of the virus suspension containing 1 × 10^5^ PFU was applied to the scarified corneas. Following this, the eyelids were closed and opened carefully several times to facilitate the absorption and distribution of the virus. A single dose of sustained release (SR) buprenorphine (1 mg/kg body weight) was injected as an analgesic after infection.

### Murine Dry Eye Disease Model

Environmental DS-induced DED was applied as previously described (33). In brief, mice were placed in a controlled environmental chamber at temperature of 21–23°C and humidity of 15% (Percival Scientific, Perry, Iowa). Airflow of 15 L/min using INTELLIS Ultra Control System and desiccant drier 50 cfm was applied. The chamber is sealed avoiding the direct exchange of air between the outside and the inside and is connected to a desiccant, which introduces air with low humidity inside the chamber. Inside the chamber, three sensors are located in order to monitor the humidity, airflow and temperature. Sensors are connected to a router in order to automatically monitor the parameters. In order to maximize exposure, mice were housed in custom-designed perforated cages (Ancare Corp. Bellmore, NY). The cages were built with vents at each side to maximize the airflow through them in order to achieve greater DS at the ocular surface. DED mice were kept under DS for 2 weeks (2 w) or 4 weeks (4 w) and then underwent subsequent experiments. Naïve control animals were housed in a normal laboratory environment with a humidity of 50–60% and a temperature of 21–23°C.

### Clinical Scoring and Tear Production

For clinical measurements, mice were anesthetized as above and 1 drop of fluorescein (Akorn Inc., Lake Forest, IL) was added to the ocular surface. Corneal fluorescein staining (CFS) scores were assessed using the National Eye Institute (NEI) scale (0–15), as previously described (34). The CFS was graded in 5 corneal regions, each ranging from 0 to 3 and the sum of the scores of all regions (range 0–15; *n* = 4/group) was measured and used for analysis. Tear secretion was measured on another set of mice using phenol red thread test (Hilco Vision Headquarters, Plainville, MA) (35, 36). The tip of the thread was placed inside the temporal eye canthus for 30 s. The wetted length was then measured to quantify the tear secretion rate.

### Histopathological Evaluation of Lacrimal Gland Infiltration

The lacrimal gland was removed and immersed for cryo-protection in sucrose 30% overnight and frozen in OCT until sectioning. Cryo-sections of 20 μm were performed, air dried up to 15 min to remove moisture and stained with 0.1% hematoxylin (Sigma; MHS-16) for 10 min in a 50 ml conical tube. Slides were rinsed in cool running distilled water for 5 min in a coupling jar. Afterwards, slides were stained with 0.5% eosin in ethanol) and dipped in distilled water. Slides were subsequently dipped alternating in 50% ethanol and 70% ethanol 10 times followed by 95% ethanol for 30 sec. Finally, slides were submerged in 100% ethanol for 1 min and washed in 100% xylene several times before imaging by microscopy. At least 10 sections per LG were evaluated morphologically by light microscopy in a masked fashion and the total number polymorphonuclear cells (PMNs) and mononuclear cells counted per section using Image J. Cells were quantified and reported as cells per millimeter square (cells/mm^2^). Five representative image per animal and 3 animals per each group were used for comparison purposes (Figure S1).

### Quantification of Lacrimal Gland Edema

To quantify LG edema, the areas between acinar cells were quantified used the software ImageJ (NIH, Bethesda, MD). The images were converted to 8-bit images. The software scanned the 8-bit image and searched for non-stained tissue. Red color was chosen to differentiate non-tissue areas. For each individual image the threshold was adjusted. In the menu, “Analyze” and then “Measure” was selected so that the area in red was quantified for each image. Percentage was determined by dividing the area quantified in red relative to the total area of the image. Three animals per group were used for comparison purposes.

### Immunofluorescence Histochemistry

Sections were fixed for 10 min in cold methanol, washed for 5 min with PBS and incubated with blocking solution constituted of 3% BSA (Sigma Aldrich, St. Louis, MO) in PBS with 0.1% of Triton-X (Sigma Aldrich). The following primary antibodies were incubated over night at 4°C to evaluate cell density in the lacrimal gland: CD45 monoclonal antibody rat anti-mouse (1:100; clone 30-F11, eBioscience San Diego, CA) and CD11c monoclonal antibody Armenian hamster anti-mouse (1:100; clone N418, eBioscience). Three washes of 10 min each with PBS were performed, followed by the secondary antibody incubation for 1 h 30 min at room temperature: TRITC- Donkey Anti-Rat (1:500; 712-025-153) and FITC-Goat Anti-Armenian Hamster (1:500; 127-095-160), respectively, both from Jackson ImmunoResearch Labs (West Grove, PA).

### Lacrimal Gland Preparation and Intravital Multiphoton Microscopy

**Figure 1:**
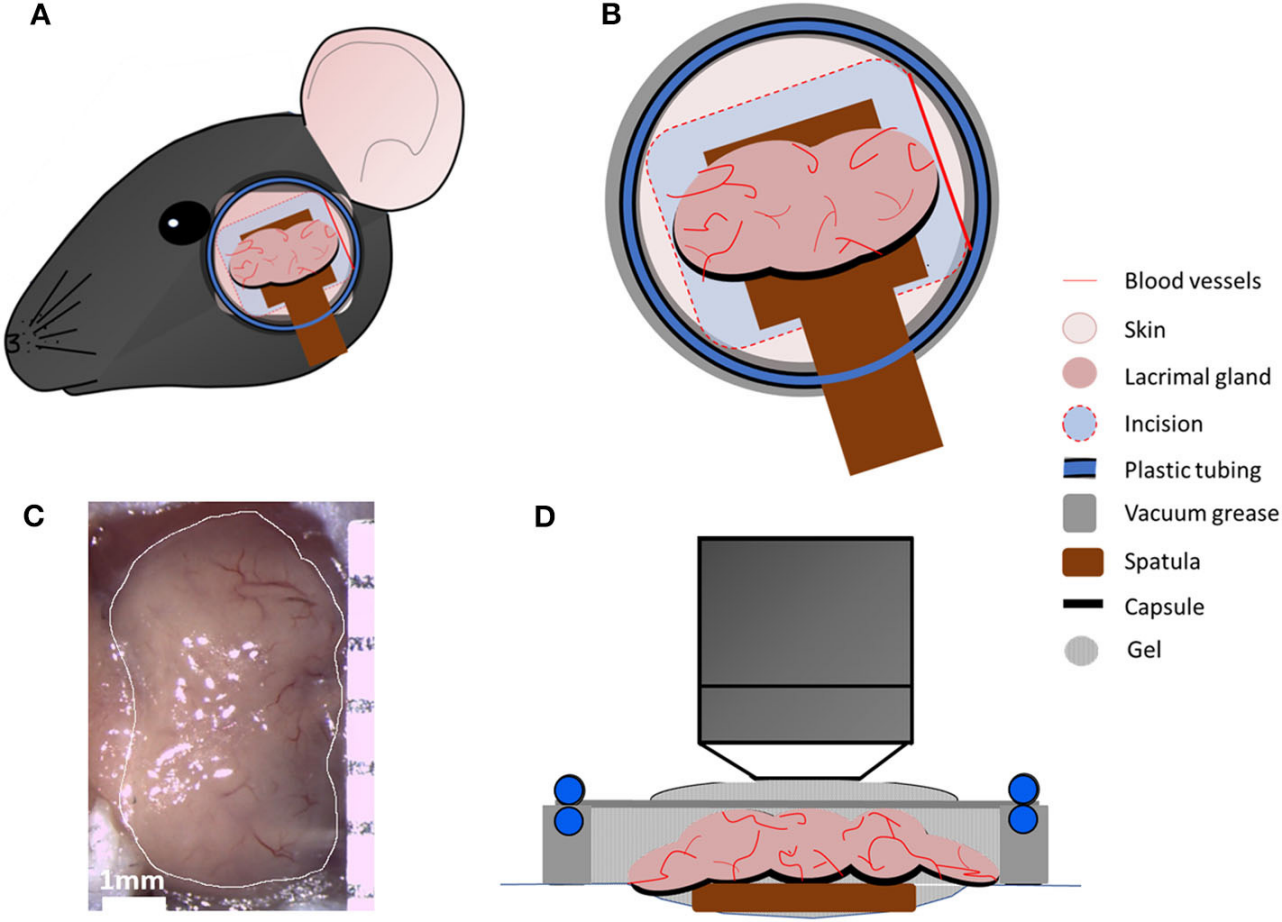
Intravital multiphoton microscopy setup. **(A)** Schematic presentation of the area between ear and eye, which was shaved, and an incision was made to expose main LG. **(B)** Schematic highlighting the en face view of the LG and the setup with a coverslip heated with circulating water allowing temperature regulation in the region as a closed chamber. **(C)** A representative image of the exposed main LG. **(D)** Schematic side view of all components of the chamber.

Animals were anesthetized by intraperitoneal injection of ketamine (100 mg/kg)/xylazine (20 mg/kg)/acepromazine (3 mg/kg) cocktail, which results in up to 75 min of deep anesthesia (21). Prior to the incision, hair between the eye and ear (~10 mm wide) was carefully removed using Nair hair removal lotion (Naircare, Princeton, NJ), followed by a single dose injection of 30 μl local analgesic (0.75% Bupivacaine HCl). A ~5 mm cutaneous incision was made 2 mm away from the eye and 3 mm away from the ear to expose the LG (Figures 1A–C). Careful removal of the soft tissues around the LG exposed the gland without damaging blood vessels. In order to stabilize the LG during imaging, a wooden spatula was placed underneath the gland. Afterwards, 5–10 μl of PBS were carefully injected into the LG capsule (the connective tissue that surrounds the lobes of the LG). This created a separation between the capsule and the lobes of the LG to enable removal of the capsule without damaging the underlying lobes.

Mice were then placed onto a custom-designed stage for LG imaging (Figure 1). In order to prevent hypothermia during imaging, the body temperature was maintained between 35 and 37°C, using a disposable hand warmer (HotHands, HearMax, Dalton, GA). The depth of anesthesia was also examined every 50–60 min and an additional dose of anesthesia cocktail was given as necessary. IV-MPM was performed using an Ultima Multiphoton Microscope System (Bruker, Fitchburg, WI) equipped with 2 MaiTai Ti/Sapphire DeepSee lasers (Newport Spectra-Physics, Irvine, CA) as previously described (21) with scanning time between 30 and 75 min and 3 μm optical section slices. Briefly, the simultaneous coaxial illumination was between 800 and 880 nm wavelengths to achieve 2-photon excitation and second harmonic generation. The laser power was set at 95, and the photomultiplier tube gain (PMTs) was set at 650 for all channels. Using a 20x-1.0 NA (Olympus XLUMPLFLN, Tokyo, Japan) water immersion objective, scans of the LG were taken every 30–60 sec during the scanning period with 512 × 512 resolution and 2-fold line averaging. Genteal ophthalmic lubricant gel (Alcon, Fort Worth, TX) was applied onto the LG and the incision to prevent desiccation during IV-MPM. High vacuum grease (Dow Corning, Midland, MI) was applied onto the bare skin surrounding the incision and additional Genteal gel was added to the area. A thin circular coverslip with tubing, for the circulation of heated water, was placed onto the exposed area in order to maintain the heat dissipation and vacuum effect as a sealed chamber during imaging. The temperature regulation during IV-MPM was conducted using a Diba polytetrafluoroethylene fitting unicon connector (Cole Palmer, Vernon Hills, IL) and polyethelene tubing, which was connected to the ring of tubing placed around the coverslip. Heated water was circulated within the tubing using a Masterflex L/S peristaltic pump (Cole Parmer, Vernon Hills, IL) with a flow rate of 30 ml/min (Figure 1B). The LG temperature (~36–37°C) was monitored throughout the IV-MPM imaging by placing a dual input digital thermometer (Omega Engineering, Stamford, CT) on the gland but 1 mm away from the imaging area in the seal chamber (Figure 1D).

### Image Analysis

In order to examine the cDC kinetics and morphology, a 4D movie was generated by importing the image stacks to the Imaris software (Bitplane, Zurich, Switzerland) as previously described (21). The motility of the transgenic fluorescent-labeled cDCs was then tracked semi-automatically in xyz positions using the 3D rendering and cell tracking function over time. 3D instantaneous velocity (cell velocity between 2 consecutive frames; μm/min), mean track speed (average velocity of a cell over time during imaging, μm/min), track length (total tracking distance of a cell, μm), displacement length (the distance of a cell traveled from start to the end of the imaging; μm) and meandering index (displacement length/track length) were calculated as previously described (21, 37). To assess 3D cell morphology, 3D surface area (μm^2^), 3D cell volume (μm^3^) and 3D sphericity (score 0–1, where 1 indicates a perfect sphere) were determined by creating a surface object for each YFP cell using the surface tool within the Imaris software.

### Flow Cytometry

Lacrimal glands were harvested, chopped and subjected to digestion for up to 30 min at 37°C in 5 mg/ml dispase (Sigma-Aldrich, St. Louis, MO), 2 mg/ml collagenase D (Roche, Indianapolis, IN) and 2 mg/ml DNAse I (MilliporeSigma, Burlington, MA) in HBSS (Thermo Fisher Scientific, Waltham, MA). The digested tissues were strained using a 70 μm nylon mesh to yield single cell suspension and the digestion was inactivated by adding RPMI supplemented with 10% FBS (Corning Inc.). After centrifugation, red blood cells were lysed using ACK (Ammonium-Chloride-Potassium) Lysing Buffer (MilliporeSigma) followed by centrifugation, and washed and resuspended in FACS buffer (Thermo Fisher Scientific).

Single cell suspensions were incubated in FACS buffer with 1% Fc receptor block (CD16/32, BioXCell, West Lebanon, NH) for 15 min to prevent non-specific binding. Samples were then stained with Live/Dead UV Blue (Thermo Fisher Scientific), and combinations of CD45 (FITC-conjugated), CD11c (BV421-conjugated), CD11b (Alexa flour F647-conjugated) or their respective isotype controls (all Biolegend, San Diego, CA) for samples from WT mice or combinations of CD45 (Pacific Blue-conjugated), CD11c (APCCy7-conjugated), CD11b (PercpCy5.5-conjugated), DCIR2 (Alexa flour-647-conjugated), Ly6G (PercpCy5.5-conjugated), NK1.1 (PECy7-conjugated), MHC class II (PerCP/Cy5.5-conjugated, clone M5/114.15.2), CD40 (PE-conjugated clone 1C10), and CD86 (PerCP/Cy5.5-conjugated clone GL-1) and their respective isotype controls (all Biolegend) for samples from CD11c^YFP^xThy^YFP^ mice for 45 min. Samples were then washed and analyzed via the BD LSR II Analyzer (BD Bioscience, San Jose, CA). Isotype controls and fluorescence minus one control were used for setting the appropriate gates in the analysis. The lists of antibodies are presented in Table S1. The sequential gating strategy for all samples, including gating on presumable immune cell population, live cells, single cells, and CD45^+^ or CD45^+^YFP^+^ cells, are presented in Figures S2–S4.

### T Cell Proliferation Assays

Splenocytes were collected from naïve WT (C57BL/6N) animals and sorted for naïve CD4^+^ T cells, identified as live CD45^+^CD3^+^CD4^+^CD44^lo^CD62L^hi^ cells (Table S1). cDCs were sorted from LGs of sham- and HSV-infected animals as live CD45^+^CD11c^+^ cells. After sorting, naïve CD4^+^ T cells were labeled with Violet Tracer following the manufacturer's protocol (CellTrace Violet Proliferation Kit, Invitrogen, Carlsbad, CA). The cDCs (5,000 cells) were cultured with naïve CD4^+^ T cells (50,000 cells) at a ratio of 1:10 in RPMI medium supplemented with 10% FBS, and cultured in 96-well round bottom plates (Corning) at 37°C. After 72 h of culture, the CD4^+^ T cells were analyzed by flow cytometry to monitor for T cell proliferation as indicated by the Violet Tracer. The tracer diffuses into cells and binds to intracellular amines, resulting in stable fluorescent staining. The co-culture with cDCs from sham-infected mice were used as unstimulated controls. On flow cytometry histograms, discrete peaks represent generation of live cells. Presented numbers on the histograms and graphs, indicate the percentage of cells undergoing at least one cycle of proliferation.

### Statistical Analysis

Statistical analyses were performed using GraphPad Prism version 8 (GraphPad Software, La Jolla, CA). Results are presented as mean and SEM. One-way ANOVA was carried out to examine the differences in the studied variables between groups with *post-hoc* comparisons using Bonferroni correction tests. Significance was set at *P* < 0.05. The Spearman rank correlation coefficient was used for correlation purposes.

## Results

### Corneal Nerve Damage Affects Dendritic Cell Function in The Lacrimal Gland

**Figure 2:**
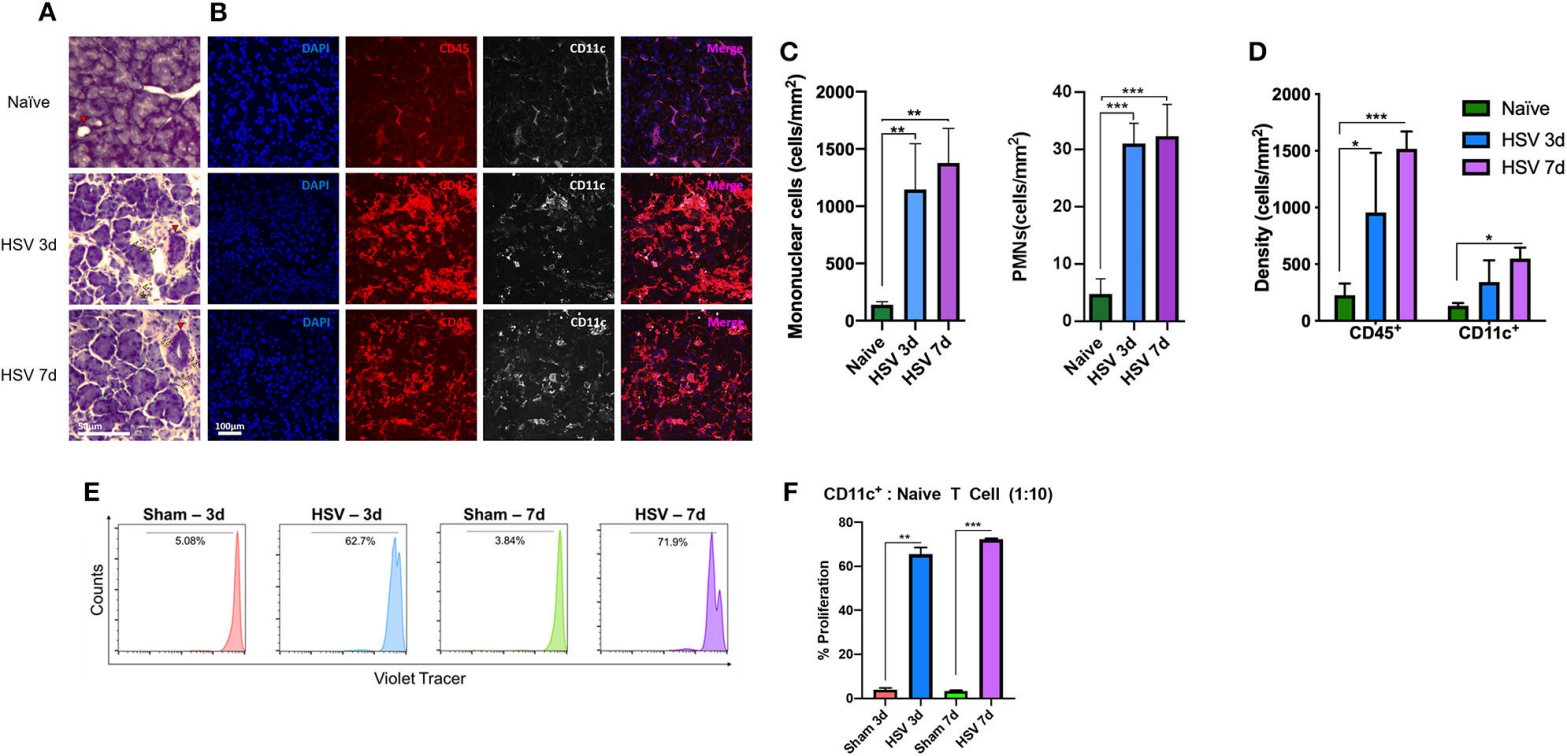
Lacrimal gland immune cell Infiltration after HSV-1 infection. **(A)** After 3- and 7- days post corneal HSV-1 infection mononuclear cells (yellow arrowhead), PMNs and duct hyperplasia (red arrowhead) is observed. **(B)** Corneal HSV-1 infection induces an increase of CD45^+^ cells and increased density in CD11c^+^ cells in the LG. **(C)** Quantification of mononuclear and PMNs cells in naïve and after 3- and 7- days post HSV-1 infection. **(D)** Quantification of CD45^+^ and CD11c^+^ cells at 3- and 7-days post- HSV-1 infection. **(E)** LG isolated CD45^+^CD11c^+^ cDCs from HSV-infected animals induce naïve T cell proliferation. Histograms are representative of T cell proliferation. **(F)** Quantification of the proliferation assay presented in **(E)**, shows the percentage of proliferating T cells. *T*-test indicates significant differences on day 3 and 7 after HSV-1 infection compared to sham-infections (*p < 0.05, **p < 0.01, ***p < 0.001).

Considering the key role of the sensory nerves as the afferent pathway of the LFU in regulating tear production, we aimed to investigate if corneal nerve damage may affect the density and function of immune cells in the LG, in particular cDCs, which serve as the professional APCs of the ocular surface (Figure 2). In order to induce corneal nerve damage we employed the clinically relevant murine model of HSV-1 keratitis, which causes severe corneal nerve damage (38). Currently, it is well-known that corneal sensory nerves affect tear production in the LG, however, it is not clear if they can affect other aspects of LG homeostasis, including immunologic state of the LG.

Histological analyses (Figure 2A) showed a profound increase in mononuclear cells and modest increase in PMNs (Figure 2C). While mononuclear cell density in naïve mice exhibited 139.6 ± 25.59 cells/mm^2^, they increased after day 3 (1,145.3 ± 400.3 cells/mm^2^, *p* = 0.008) and day 7 post-infection (1,377.2 ± 302.1 cells/mm^2^, *p* = 0.003). PMN density in naïve mice was 4.4 ± 2.9 cells/mm^2^, and significantly increased after 3 days (30.7 ± 3.8 cells/mm^2^) and 7 days (32.0 ± 5.9 cells/mm^2^; *p* < 0.001 for both; Figure 2A) post HSV-1 infection. H&E staining indicated increased LG edema. While naïve mice displayed 5.5 ± 2.4% area between acini, edema significantly increased after day 3 (29.7 ± 3.7%) and day 7 (34.2 ± 4.3%, *p* < 0.01 for both; Figure S1A, red area and Figure S1B) post HSV infection. Further, signs of duct hyperplasia and fibrosis were observed (Figures 2A, S1A red arrowhead and delineated by a green dashed area, respectively). Using immunofluorescence histochemistry we observed that compared to naïve mice (179.3 ± 66.6 cells/mm^2^), CD45^+^ cell density significantly increased after 3 and 7 days of HSV-1 infection to 956.5 ± 525.6 cells/mm^2^ (*p* = 0.003) and 1,517.5 ± 153.5 cells/mm^2^ (*p* < 0.001; Figures 2B,D), respectively. In line with the results of the CD45^+^ cell density, CD11c^+^ cell density also increased; while naïve mice exhibited 149.6 ± 13.6 cells/mm^2^ after 3d and 7d of HSV infection, the density was 343.3 ± 190.3 cells/mm^2^ (*p* = 0.88) and 451.7 ± 165.4 cells/mm^2^ (*p* = 0.04), respectively (Figures 2B,D).

Thus, we next aimed to assess if damage to the ocular surface sensory nerves may affect functionality of cDCs within the LG. After 3- and 7-days post-infection (dpi), we sorted cDCs from the LGs and co-cultured them with naïve CD4^+^ T cells in order to evaluate if cDCs could induce T cell proliferation. We found that while cDCs sorted from sham-infected mice could induce minimal T cell proliferation (Figure 2E), cDCs from HSV-1 infected mice induced considerable T cell proliferation as ~62.7 and 71.9% of T cells co-cultured with cDCs sorted from HSV-1 infected mice on 3 and 7 dpi underwent at least one cycle of proliferation (Figures 2E,F).

### Lacrimal Gland Conventional Dendritic Cells Are Increased During Dry Eye Disease

**Figure 3:**
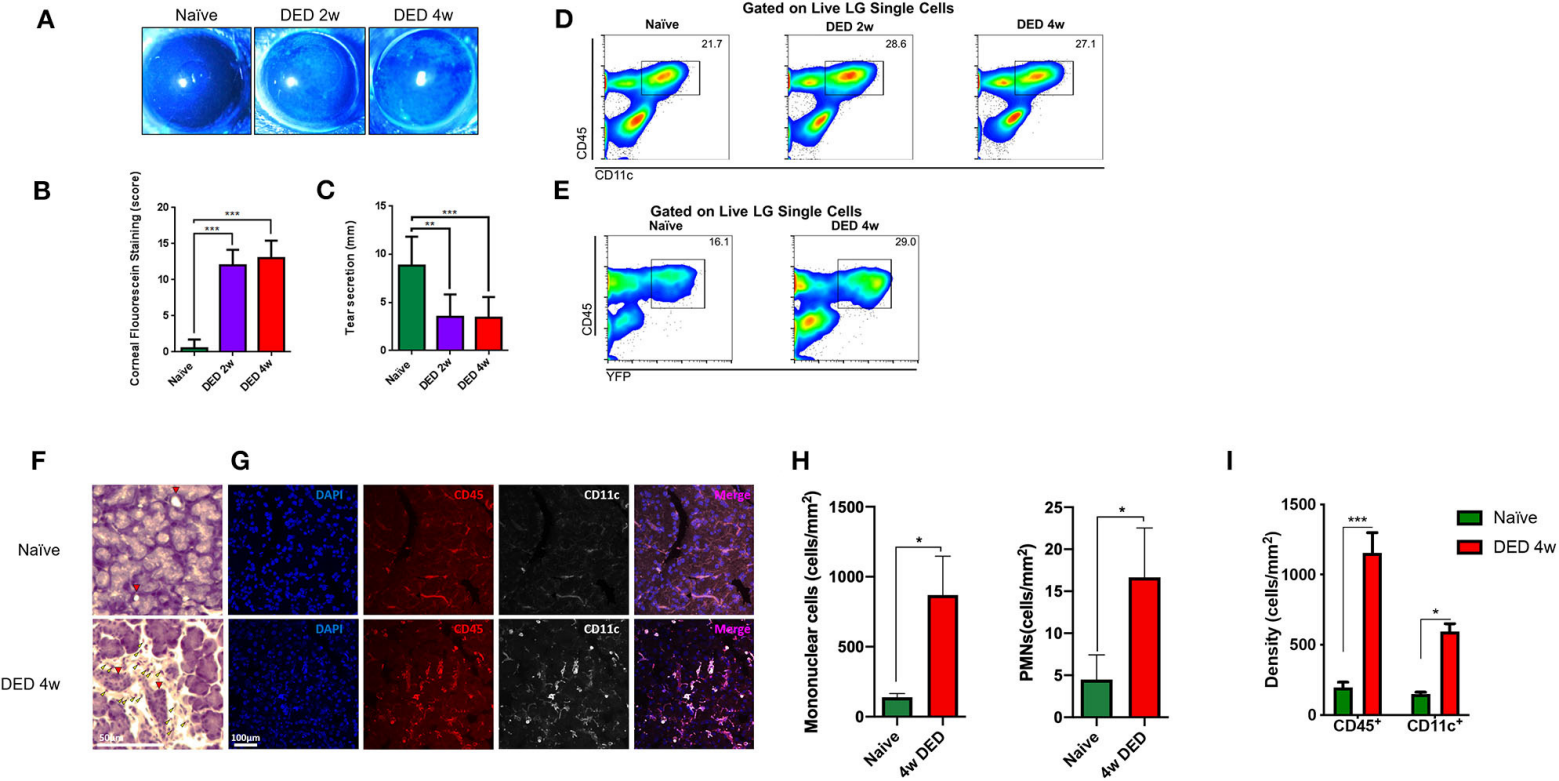
Clinical changes and alterations in conventional dendritic cell density of the lacrimal gland in dry eye disease. **(A)** Representative images of fluorescein staining. **(B)** Quantification of CFS (*n* = 4/group). **(C)** Quantification of the tear secretion using phenol-red thread test (*n* = 4/group). *p*-values were calculated using one-way ANOVA followed by the Bonferroni multiple comparison test **p < 0.01, ***p < 0.001. **(D)** Percentages of CD45^+^CD11c^+^ cells in the LG naïve state as well as after 2 and 4 w exposure to DS in C57BL/6N animals. **(E)** Percentage of CD45^+^YFP^+^ cells in the LG of CD11c^YFP^×Thy1^YFP^ transgenic mice in naïve state and after 4 w exposure to DS. **(F)** LG histopathology of naïve and after 4 w of DED (yellow arrowhead indicates infiltrating mononuclear cells, red arrowhead indicated LG ducts). **(G)** Immunofluorescence of CD11c^+^ and CD45^+^ cells within the LG in naïve and 4 w of DED. **(H)** Quantification of mononuclear cells and PMNs in naïve and 4 w of DED. **(I)** Quantification of the cell density presented in **(G)** as cells/mm^2^ (*p < 0.05, ***p < 0.001).

Having established the potential importance of corneal innervation in LG immune homeostasis and functionality of cDCs in this tissue, we next examined if DS, which generally causes milder corneal sensory nerve damage (9) than HSV keratitis, but is more prevalent clinically, has an impact on the density of cDCs in the LG after DS. Greater CFS score (Figure 3A) and lower tear secretion were observed in CD11c^YFP^×Thy^YFP^ mice exposed to DS compared to naïve controls. The CFS score was 12.0 ± 3.5 at 2 w and 13.0 ± 4.5 at 4 w, compared to naïve mice (1.0 ± 1.5; *p* ≤ 0.001; Figures 3A,B). Tear secretion in naïve mice was 9.0 ± 4.5 mm, whereas it was reduced to 3.0 ± 2.5 mm at 2 w and remained unchanged (3.0 ± 2.0 mm) at 4 w of exposure to DS (*p* ≤ 0.004; Figure 3C). Thus, consistent with previous reports, our DS chamber induces decreased tear volume and increased CFS, two hallmarks of DED, as early as 2 weeks.

We next performed flow cytometric analysis on the LGs of naïve and DED C57BL/6N mice. As shown in Figure S2, forward and side scatter, as well as viability dye were used to gate on presumed population of immune cells (~2% of total single cells suspension) by the exclusion of debris (Figure S2A), dead cells (Figure S2B), and doublets (Figure S2C). We next gated on CD45^+^ and CD11c^+^ (Figure 3D) to analyze all cDCs in the LG. As depicted in Figure 3D, the density of CD45^+^CD11c^+^ cDCs after 2 and 4 w of DS was higher than the population in naïve mice. Next, we assessed if YFP^+^ cells in our transgenic CD11c^YFP^xThy^YFP^ mice showed a similar pattern after DS in the LG. Following sequential gating on presumed population of immune cells by excluding debris (Figure S3A), dead cells (Figure S3B), and doublets (Figure S3C), we observed that 16.1% CD45^+^YFP^+^ cells were found in the naïve LG, while at 4 w of DS this population was increased to 29.0% (Figure 3E and Figure S3). This population was also positive for CD11c, CD11b, DCIR2, and negative for Ly6G and NK1.1 (Figure S4), confirming their cDC phenotype. Importantly, CD45^+^CD11c^+^ cDCs, after 4 w of DED, exhibited a 1.42-fold increase of the co-stimulatory marker CD40 (*p* < 0.05; Figure S5). In contrast, no significant changes were observed for MHC-II and CD86 on LG cDCs after DED.

Furthermore, histological analyses exhibited increased mononuclear cells after 4 w of DED (869.9 ± 277.2 cells/mm^2^, *p* = 0.063 Figures 3F,H yellow arrowhead) compared to naïve mice and PMNs were also increase after 4 w of DED (16.6 ± 5.9 *p* = 0.057). Edema after 4 w of DED increase to 23.1 ± 0.6% (*p* < 0.01; Figures S1A,B red area). Further, CD45^+^ cells increase after 4 w of DED compared to naïve mice (1,157.8 ± 70.2 vs. 179.3 ± 66.6 vs. cells/mm^2^; *p* < 0.001; Figures 3G,I). While CD11c after 4 w of DED compared to naïve mice was 594.4 ± 55.8 cells/mm^2^ vs. 149.6 ± 13.6 cells/mm^2^, respectively (*p* < 0.05, Figures 3G,I). H&E staining further indicated increased LG edema. While naïve mice displayed 5.5 ± 2.4% area between acini, edema significantly increased after 4 w of DED to 23.1 ± 0.6% (*p* < 0.01; Figures S1A,B red area). In addition, signs of fibrosis were observed (Figure S1A first row; green dashed area). Collectively, our findings show that DS-induced DED is accompanied by increased density of cDCs in the LG.

### Intravital Multiphoton Microscopy of The Lacrimal Gland

Having shown that DED results in increased cDC density in the LG, we next aimed to study the morphological alterations and kinetic changes of LG cDCs by IV-MPM. Morphologic and kinetic alterations of naïve cDCs were compared with DED-induced inflammation of the LG.

### Analysis of Morphologic Parameters

**Figure 4:**
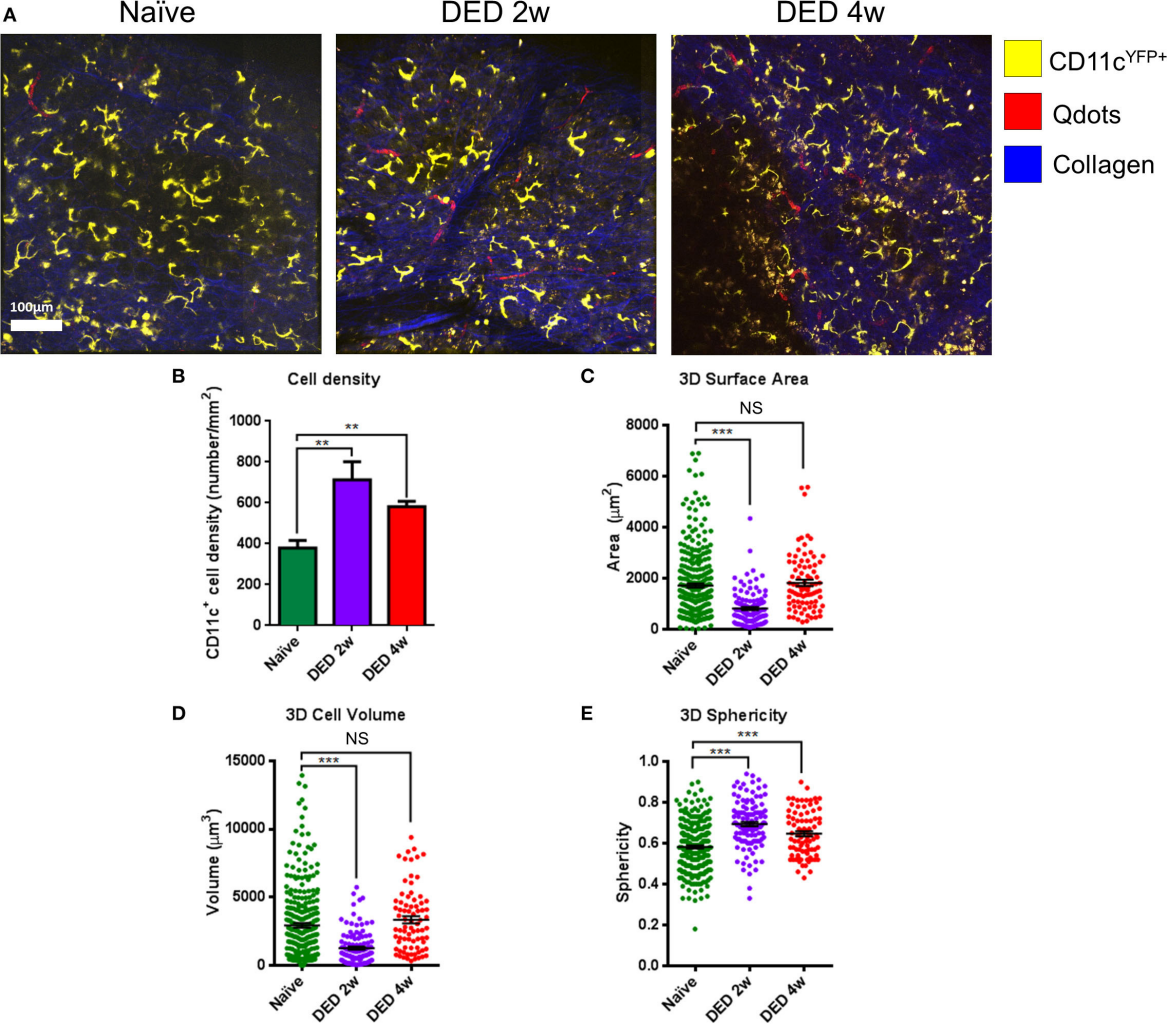
Morphological alterations in lacrimal gland conventional dendritic cells during dry eye disease. **(A)** Representative IV-MPM images of cDCs in the LG of CD11c^YFP^×Thy1^YFP^ mice in naïve state (left panel), and at 2 w (middle panel) and 4 w (right panel) of exposure to DS. Note increased density and sphericity of cells after exposure to DS. Scale bar: 100 μm. **(B)** Density of cDCs in transgenic CD11c^YFP^×Thy1^YFP^ mice. **(C–E)** 3D morphologic analyses, including **(C)** 3D surface area, **(D)** 3D cell volume, and **(E)** 3D sphericity. Results are presented as mean ± SEM. One-way ANOVA (upper right) and Bonferroni multiple comparison test. **p < 0.01, ***p < 0.001.

The analysis of cDC distribution in naïve CD11c^YFP^×Thy^YFP^ mice demonstrated a range of cell sizes among cDCs. *In vivo* analysis of cDCs revealed uniform distribution of cDCs in the LG lobes in both naïve (Video S1) and after 2 w (Video S2) and 4 w (Video S3) of exposure to DS (Figure 4A). However, the cDC density in naïve mice was 379.2 ± 38.2 cells/mm^2^, while 2 and 4 w of DS was 712.5 ± 88.4 and 581.2 ± 26.5 cells/mm^2^, respectively (Figure 4B). The data show that DS induced a robust increase in DC density at 2 w, which became slightly less pronounced at 4 w, once the acute phase had subsided. These new results on CD11c density were correlated with our initial findings by immunofluorescence histochemistry that demonstrated a cDC density 149.6 ± 13.6 cells/mm^2^ in naïve mice and 594.4 ± 55.8 cells/mm^2^ after 4 w of DED (*p* < 0.01, for naïve *r* = 0.94; for DED *r* = 0.95). This increased number of cDCs after DS compared with naïve animals was previously corroborated by flow cytometry. To have a better understanding of the morphological changes of cDCs after DS we analyzed the cell surface area, volume and sphericity of cDCs at 2 and 4 w after DS. Interestingly, 3D surface area and 3D cell volume were decreased after 2 w of DS compared with steady state. cDCs after 2 w of DS had a 3D cell surface of 809.3 ± 60.4 μm^2^ compared with naïve mice showing a cDC surface area of 1,717.4 ± 79.4 μm^2^. In addition, the 3D cell volume was 2,929.3 ± 159.4 μm^3^ in naïve mice, and 1,274.0 ± 112.3 μm^3^ at 2 w post DS (one-way ANOVA with Bonferroni *post-hoc* test *p* < 0.001) (Figures 4C,D). In contrast, we found no difference in 3D cell surface area 1,717.4 ± 79.4 vs. 1,819.1 ± 121.8 μm^2^ (*p* = 0.77) and 3D cell volume 2,929.3 ± 159.4 vs. 3,340.0 ± 260.6 μm^3^ between naïve and 4 w of DED (*p* = 0.37; Figures 4C,D). However, the 3D sphericity of cDCs was increased at 2 w (0.694 ± 0.011) and 4 w (0.647 ± 0.012) after DS, compared with naïve mice (0.581 ± 0.007, *p* < 0.001 for both comparisons) (Figure 4E). The decreased size and volume, together with the increased sphericity suggest a more migratory phase of cDCs at 2 w during the active phase of inflammation. At 4 w, cell size and volume increase and cDCs show decreased sphericity, demonstrating a more sessile phase during persistent inflammation. Altogether, these results support that there is a shift in the dendritic state (soma and dendrites) characteristic of resting cDCs toward a more dynamic state (round shape with few to no dendrites).

### Kinetics and Motility Parameters of Conventional Dendritic Cells in The Lacrimal Gland

**Figure 5:**
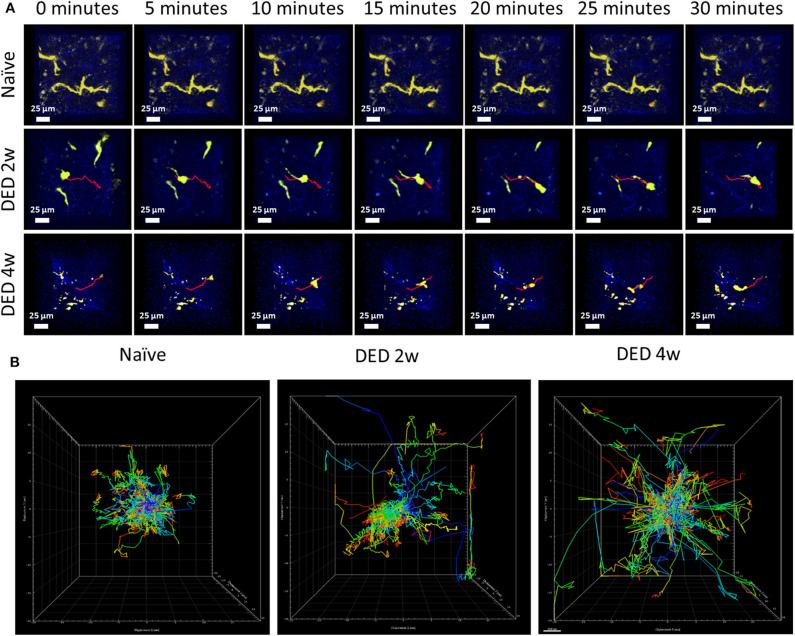
Conventional dendritic cell motility in the lacrimal gland using IV-MPM during dry eye disease. **(A)** Representative en face time course images of cDCs in the LG of CD11c^YFP^×Thy1^YFP^ mice in naïve mice (21), and mice exposed to DS for 2 w (middle) and 4 w (bottom) over a 30 min time period reveal differences of kinetic and morphological changes of cDCs. Scale bars, 25 μm. **(B)** Representative centered kinetic tracks of cDCs, shows displacement and directionality of the cells in naïve mice and after DS. During naïve state (left panel), the tracks were shorter with sampling movements and no directionality. After exposure to DS (2 w in middle panel, 4 w in right panel), cDCs become more motile with larger tracks with no preferential direction.

Having shown 3D morphological alterations in LG cDCs, we next aimed to analyze if kinetics of cDCs (including 3D instantaneous velocity, mean track speed, track length and displacement length) are altered *in vivo*, following exposure to DS using the transgenic CD11c^YFP^×Thy^YFP^ mice. Importantly, previous work has shown that temperature is a critical parameter, since leukocyte function, including kinetics, can be modulated by temperature (23, 39, 40). Thus, in this work, we held the physiological temperature (37°C) during the entire imaging sequence. While we only observed sampling movements of cDCs with minimal displacement in naïve mice (Video S1), at 2 w (Video S2) and 4 w (Video S3) of DS, we observed a considerable alteration in cDC motility, showing more locomotion with longer tracks (Figure 5A; a sample track in each panel is represented in red). As depicted in Figure 5B, cDCs exposed to DS showed increased motility in all directions (x, y, and z).

**Figure 6:**
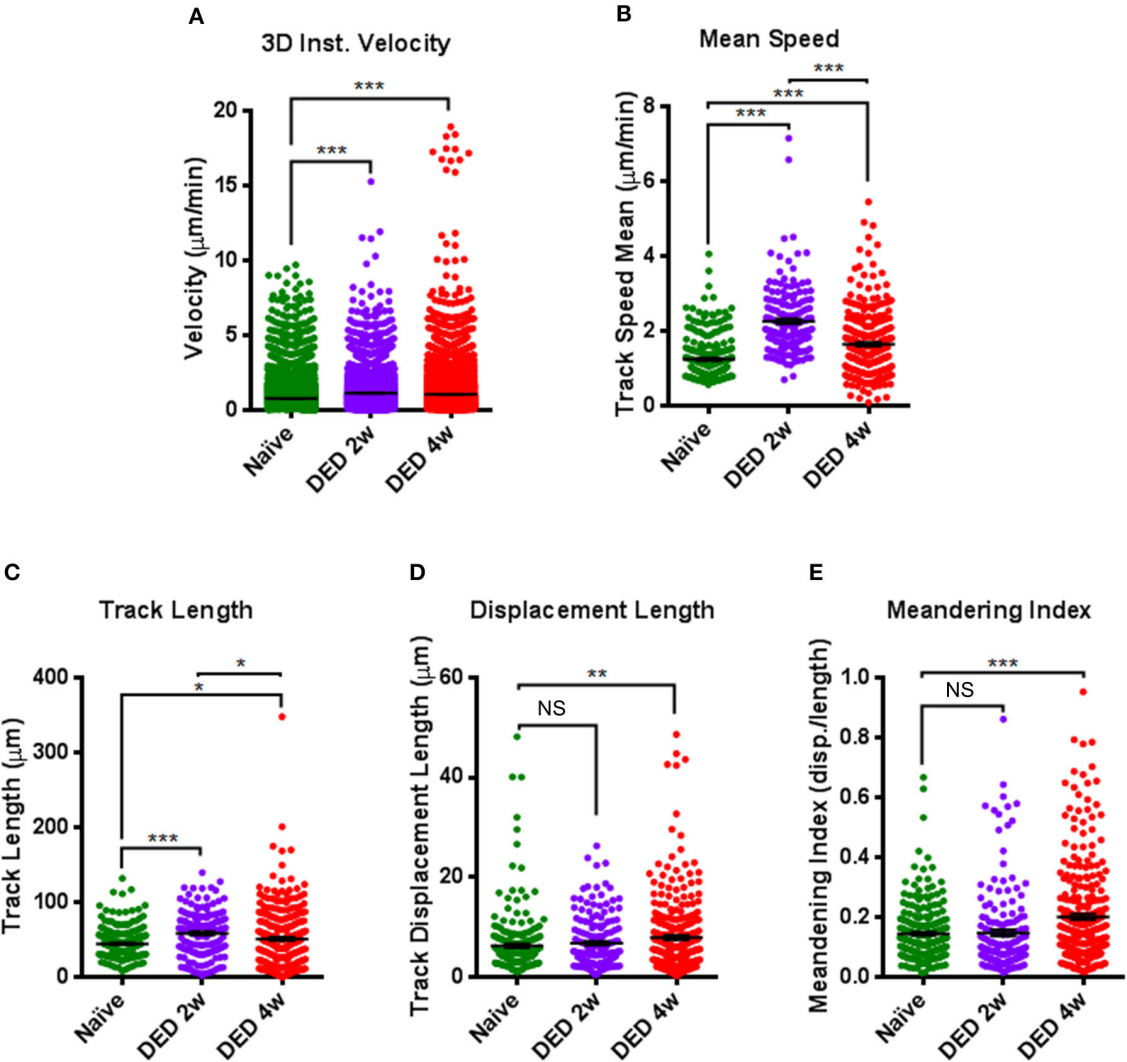
Comparison of kinetic parameters of conventional dendritic cells in the lacrimal gland using IV-MPM during dry eye disease. Analyses of the kinetics of cDCs, including **(A)** 3D instantaneous velocity, **(B)** track speed mean, **(C)** track length, **(D)** displacement length, and **(E)** meandering index. Results are presented as mean ± SEM. One-way ANOVA (upper right) and Bonferroni multiple comparison test. *p < 0.05, **p < 0.01, ***p < 0.001.

Further, after both 2 w (1.135 ± 0.015 μ/min) and 4 w (1.052 ± 0.011 μ/min) following DS, we showed increased 3D instantaneous velocity compared with cDCs from naïve animals (0.771 ± 0.007 μ/min, *p* < 0.001 for both comparisons; Figure 6A). The mean track speed of cDCs was also increased at 2 w (2.257 ± 0.069 μ/min) and 4 w (1.645 ± 0.049 μ/min) compared with naïve state (1.265 ± 0.044 μ/min, *p* < 0.001 for both comparisons; Figure 6B). Interestingly, mean track speed of cDCs at 2 w of exposure to DS was significantly higher compared with cDCs at 4 w of exposure to DS (*p* < 0.001; Figure 6B).

We next examined the changes in track length and displacement length of cDCs in the LG after exposure to DS. Quantifications of these parameters revealed that track length was 44.49 ± 1.22 μm in naïve mice, which was significantly augmented at both 2 w (58.31 ± 2.22 μm, *p* < 0.001) and 4 w (50.78 ± 2.27 μm, *p* = 0.041) after DS (Figure 6C). Similar to mean track speed, cDCs at 2 w of exposure to DS, exhibited longer track lengths compared with cDCs after 4 w of exposure (*p* = 0.034; Figure 6C). In contrast, we only found significant changes in the displacement length of cDCs in naïve mice (6.27 ± 0.37 μm) compared to cDCs in mice exposed to DS for 4 w (7.88 ± 0.43 μ, *p* = 0.009; Figure 6D).

Moreover, while we did not observe a significant difference in the meandering index of cDCs at 2 w following exposure to DS (0.147 ± 0.011, *p* = 0.98) compared with cDCs in naïve mice (0.144 ± 0.006), at 4 w after DS, cDCs demonstrated a significant increase, but still relatively low meandering index (0.201 ± 0.010, *p* < 0.001; Figure 6E), suggestive of a random walk pattern. Collectively, we observed considerable alterations in all kinetic parameters of cDCs after exposure to DS compared to naïve mice.

## Discussion

In the present work, we establish a novel model of lacrimal gland IV-MPM imaging to study 3D morphology and kinetic properties of cDCs in the LG after DS-induced DED. We demonstrate that the cDC density in the LG is higher during DED, and that cDCs become smaller, more spherical, but more motile in DS-induced DED compared to naïve mice. Histopathological and immunofluorescence histochemistry analyses, showing the increased density of LG mononuclear cells, CD45^+^ leukocytes, as well as CD11c^+^ cDCs after both DED and HSV-1 keratitis, confirm our findings as observed by IV-MPM. Altogether, these results suggest that the alterations of cDCs are, at least in part, due to the damage of the sensory nerves on the ocular surface.

DED is a multifactorial disease, and DS is one of the risk factors initiating inflammatory responses at the ocular surface resulting in DED. It has been shown that DS causes reduced tear break-up time and LG dysfunction (17, 41). It is thought that DS contributes to DED pathophysiology by damaging the ocular surface and corneal sensory nerves. Subsequently, the damage to corneal nerves, which comprises the afferent pathway of the LFU, leads to low tear secretion via diminished signaling to the parasympathetic nerve fibers (42). However, the mechanism underlying damage to the corneal sensory nerves on LG immune cell populations is unknown. The LG is mainly responsible for tear secretion through a tightly regulated process incorporating all components of the LFU. Innervation of the ocular surface and the lacrimal gland have been shown to be altered in mice with DED, as compared to naïve mice. Several murine models of DED, including DS-induced DED (43, 44), DS combined with scopolamine to induce suppression of tear production (9), a model in which the LG is removed (45), an experimental autoimmune lacrimal keratoconjunctivitis model (46), as well as CD25^−/−^ and Aire^−/−^ mice that develop Sjögren's syndrome (47, 48) have all demonstrated significantly decreased corneal sub-basal nerve density compared with their respective control mice, irrespective of the model used. Further, in TSP^−/−^ mice that present with aqueous deficient DED, the neuronal innervation of the LG, and in particular the parasympathetic nerves, are substantially decreased when compared to lacrimal glands of age-matched WT mice (49). We show that the density of LG immune cells, in particular cDCs, is increased after nerve damage. Further, the destruction of corneal nerves after corneal HSV-1 infection enhances cDCs capacity in stimulating T cell proliferation. Along with changes in cDC activation, structural changes of the LG after HSV infection are observed with increased edema and increase in fibrosis. Therefore, our results indicate that corneal sensory nerves, as the afferent pathway of the LFU, directly or indirectly modulate the immune cell density and function in the LG. Nonetheless, while DED causes less severe nerve alterations on the ocular surface, HSV-1 infection results in more severe nerve injury (50, 51). However, it is important to note that several factors can contribute to both the nerve and cDC changes observed during HSV-1 keratitis. These include, the severe neurotrophic effect resulting in ocular surface damage, increased inflammation, and the effect of the HSV-1 itself. Thus, while the decrease in nerves during HSV-1 keratitis is more severe than in DED, we cannot rule out an effect from these additional factors that result in morbidity. For example, a recent study has used different strategies to deplete immune cell subpopulations to dissect the contribution of the virus to nerve damage. Reports show higher levels of nerve damage after depletion of cDCs and/or macrophages in PSGL-1^−/−^ mice that prevent *de novo* immune cell recruitment (32), suggesting that the virus itself and not inflammation largely contribute to nerve damage observed during HSV-1 keratitis. Further, increased pro-inflammatory cytokines and chemokines (52–54), changes in neuropeptides (55), as well as leukocyte recruitment, such as T cell activation and recruitment, have been demonstrated in HSV-1 keratitis (56). In addition, viral particles along with CD4^+^ and CD8^+^ T cell infiltration have been found within the LG after HSV-1 infection (57). Thus, alterations in cytokines and chemokines may, in part, explain the more pronounced infiltration of immune cells in the LG after HSV-1 keratitis, as compared to DED.

Based on the literature, inflammation of the ocular surface and corneal sensory nerve damage are observed in mice exposed to DS and individuals with DED (58, 59). The LG cDCs density increases as early as 2 w after DS and remains elevated up to 4 w of exposure to DS. This effect could in part be explained by increased expression of the pro-inflammatory cytokines IL-1, IL-6, IL-17, IL-23, TNF-α, and IFN-γ that have previously been shown in the LG at 2 and 4 weeks after exposure to DS (9). Thus, cDC density, kinetics and morphological changes presented in our work, could in part be driven by differential expression of these pro-inflammatory cytokines. In line with our findings, a recent study showed that CD11b^+^ myeloid cells were recruited to the LG at 2 and 4 weeks after exposure to DS (8). The increased density of cDCs and myeloid cells can be explained by the recruitment of circulating cDCs and/or monocytes, which can locally differentiate into cDCs (60, 61). However, this experiment did not examine if the increased density of cDCs is due to potential proliferation of the resident cDCs in the LG or from the infiltrating myeloid-derived cells.

cDCs are highly functional immune cells, inducers of dynamic processes of inflammation and/or tolerance (62), they actively shift their morphology in peripheral tissues such as skin (63) and cornea (21) during inflammation. In recent years, the initiation of immune responses and/or maintenance of central and peripheral tolerance have been studied via cDC kinetics, morphological features, and their interaction with T cells using intravital imaging (27, 64, 65). In this study, we show that LG cDCs are dendritiform with low sphericity during steady state, whereas following DED, they become less dendritiform and more spherical, and demonstrate early signs of activation, as the increased expression of the co-stimulatory marker CD40 shows. This finding is consistent with the previous reports showing that the peripheral cDCs in the naïve setting showed dendritiform morphology with increased surface area to maximize surveillance of the tissue microenvironment. During inflammation, their dendrites are gradually retracted, leaving them with fewer and shorter processes and more spherical cell bodies to facilitate their migration in tissues. While resident populations of cDCs within the skin and gut epithelium are sessile and dendritiform, after different stimuli, such as LPS administration or *Salmonella typhimurium* infection, spherical cells have been demonstrated to become predominant (66, 67). In addition, Linquist et al. have shown that in lymph nodes, LPS-activated cDCs become more motile (27). Furthermore, we have recently shown kinetics and morphological alterations of immune cells following acute sterile inflammation within the cornea, demonstrating that MHC class II^+^ cells become more spherical, with increased velocity and larger displacement following inflammation (21). Similarly, in the current work, after inflammation induced by DS, LG cDCs exhibited reduced cell surface, become smaller, acquire a spherical shape, and become motile. The morphological changes during inflammation can also be observed in monocytes, macrophages, T cells, and natural killer cells during inflammation.

Our results show that naïve cDCs in LG are sessile with a low mean speed, while their motility increased after 2 w exposure to DS and remained elevated, although to a lesser extent, at 4 w. Previous studies have demonstrated that activated cDCs within lymph nodes exhibit a mean speed of ~3 μm/min during inflammation compared to the resident naïve cDCs which had a mean speed of ~1 μm/min (27). Recently, our group has shown that the mean speed of corneal cDCs was higher following acute thermal injury compared to the naïve setting (21). The differences in mean speed of cDCs reported in the literature and our study could be due to the differences between the tissue microenvironments. This tissue-specific effect is also supported by reports showing that mean speed of cDCs was 6.6 μm/min in paracortical areas of the popliteal lymph nodes (26) and 5.9 ± 1.0 μm/min in explanted lymph nodes (68).

Naïve LG cDCs exhibit sampling movements in concordance with their immature state. This, in part, can be explained by an active role to maintain immune tolerance, also seen within the cornea (21) and epidermis (43). Although cDCs become more motile, as indicated by longer track and displacement lengths after 2 w and 4 w of DS-induced DED compared to controls, we observe no preferential directionality in either time point, suggestive of a random walk pattern. This is consistent with our previous study on corneal cDCs in naïve state and during acute inflammation, in which cDCs exhibit random movements as well (21).

Recently, several studies have shown that the peripheral nervous system (PNS), in addition to mediating communication between the central nervous system and peripheral tissues, also controls innate immune responses via non-specific responses to pathogens (69, 70). However, dysfunction or damage to the PNS may in contrast mediate pro-inflammatory innate responses, called “neurogenic inflammation” (71–74). In addition, sympathetic nerves have been shown to regulate leukocyte homing to tissues (75, 76). Moreover, both sympathetic and sensory innervation of the skin, lung and gut have been shown to influence cDC migration and motility (77, 78). Thus, neurogenic inflammation secondary to DS can result due to dysfunction of ocular surface nerves, resulting in decreased tear production, and alterations of the autonomous nervous system, leading to LG inflammation. A limitation of our study is the lack of detailed mechanistic and signaling data, demonstrating the direct effect of corneal nerve changes on the LG. We hope that the current intravital imaging model of the LG, together with the data provided, will enable and stimulate future research in this important area.

In summary, herein we show a newly developed methodology to study LG immune cell kinetics and 3D morphology in a transgenic murine model by using intravital multi-photon imaging. By using IV-MPM, the spatiotemporal organization of cDCs in the LG of naïve and DS-induced DED can be investigated. We have provided evidence that damage to corneal sensory nerves modulates the immune responses in the LG. This corneal nerve damage resulting from DS could explain the altered cDC kinetics and morphology within the LG. Thus, this study demonstrates that intravital multi-photon imaging offers opportunities for studying *in vivo* immune cell kinetics in diseases affecting the LG.

## Data Availability Statement

The raw data supporting the conclusions of this article will be made available by the authors, without undue reservation, to any qualified researcher.

## Ethics Statement

The animal study was reviewed and approved by Tufts Department of Lab Animal Medicine.

## Author Contributions

GO, CC, YS-R, AJ, BK, DH, DZ, and PH designed the research. GO, AJ, BK, and DH performed the research. CC analyzed the MPM movies. GO, CC, and AJ drafted the manuscript. YS-R, BK, DH, DZ, and PH edited the manuscript. All authors contributed to the article and approved the submitted version.

## Conflict of Interest

The authors declare that the research was conducted in the absence of any commercial or financial relationships that could be construed as a potential conflict of interest.

## Supplementary Material

Figure S1 LG histopathology in naïve mice, 3 and 7 days post HSV-1 infection, and 4w post DED. **(A)** Converted images of LG histopathology using ImageJ software of all experimental groups. The red extent exhibits the area between acinar cells in all groups. **(B)** Quantification of the area between acinar cells expressed in percentage of area in red of total image area. Results are presented as mean ± SEM. One-way ANOVA and Bonferroni multiple comparison test. ^**^*p* < 0.01, ^***^*p* < 0.001.

Figure S2 Sequential gating strategy for flow cytometry on the lacrimal gland in naïve and after 2 and 4 w of exposure to DS in WT C57BL/6N mice. **(A)** SSC-A and FSC-A of LG single cell suspension, showing gating out debris and gating on presumable population of immune cells. **(B)** Gating on live cells. **(C)** Gating on single cells. **(D)** Gating on CD45^+^ immune cells among live single cells.

Figure S3 Sequential gating strategy for flow cytometry on the lacrimal gland in naïve and after 4 w exposure to DS in CD11c^YFP^×Thy1^YFP^ mice**. (A)** SSC-A and FSC-A of LG single cell suspension, showing gating out debris and gating on presumable population of immune cells. **(B)** Gating on live cells. **(C)** Gating on single cells. **(D)** Gating on double positive CD45^+^YFP^+^ cDCs among live single cells.

Figure S4 Flow cytometric characterization of cells in the lacrimal gland of CD11c^YFP^×Thy1^YFP^ mice. Flow cytometry histograms showing expression of CD11c, CD11b, DCIR2, Ly6G, and NK1.1 markers on CD45^+^YFP^+^ cells reveals their identity as cDCs. Light gray histograms show fluorescent minus one isotype controls.

Figure S5 Flow cytometric characterization of conventional dendritic cells in the lacrimal gland after 4 weeks of exposure to desiccating stress. Histograms showing expression of MHC-II, CD86, and CD40 on CD45^+^CD11c^+^ cells reveal that in cDCs, CD40 is increased after 4w of DED compared to naïve mice. Light gray histograms show fluorescent minus one isotype controls.

Video S1 Representative IV-MPM movie of lacrimal gland in a naïve transgenic CD11c^YFP^×Thy1^YFP^ mouse. The video highlights sampling movement of cDCs with minor displacement in the naïve lacrimal galnd. Blue, second harmonic generation delineating collagen, yellow, yellow fluorescent protein expressed by cDCs.

Video S2 Representative IV-MPM of lacrimal gland in a transgenic CD11c^YFP^×Thy1^YFP^ mouse after 2 weeks of exposure to desiccating stress. The video demonstrates the spherical cDCs with fewer dendrites which move long distances in the lacrimal gland. Blue, second harmonic generation delineating collagen, yellow, yellow fluorescent protein expressed by cDCs.

Video S3 Representative IV-MPM of lacrimal gland in a transgenic CD11c^YFP^×Thy1^YFP^ mouse after 4 weeks of exposure to desiccating stress. The video shows elevated density of cDCs with spherical shape which travel with a high speed in the lacrimal gland. Blue, second harmonic generation delineating collagen, yellow, yellow fluorescent protein expressed by cDCs.

Table S1 Antibody list.
