# Supplementary figures and images for: Effect of Dry Eye Disease on the Kinetics of Lacrimal Gland Dendritic Cells as Visualized by Intravital Multi-Photon Microscopy
Source: Front Immunol. 2020 Aug 12;11:1713. doi: 10.3389/fimmu.2020.01713 (PMC7434984; doi:10.3389/fimmu.2020.01713)

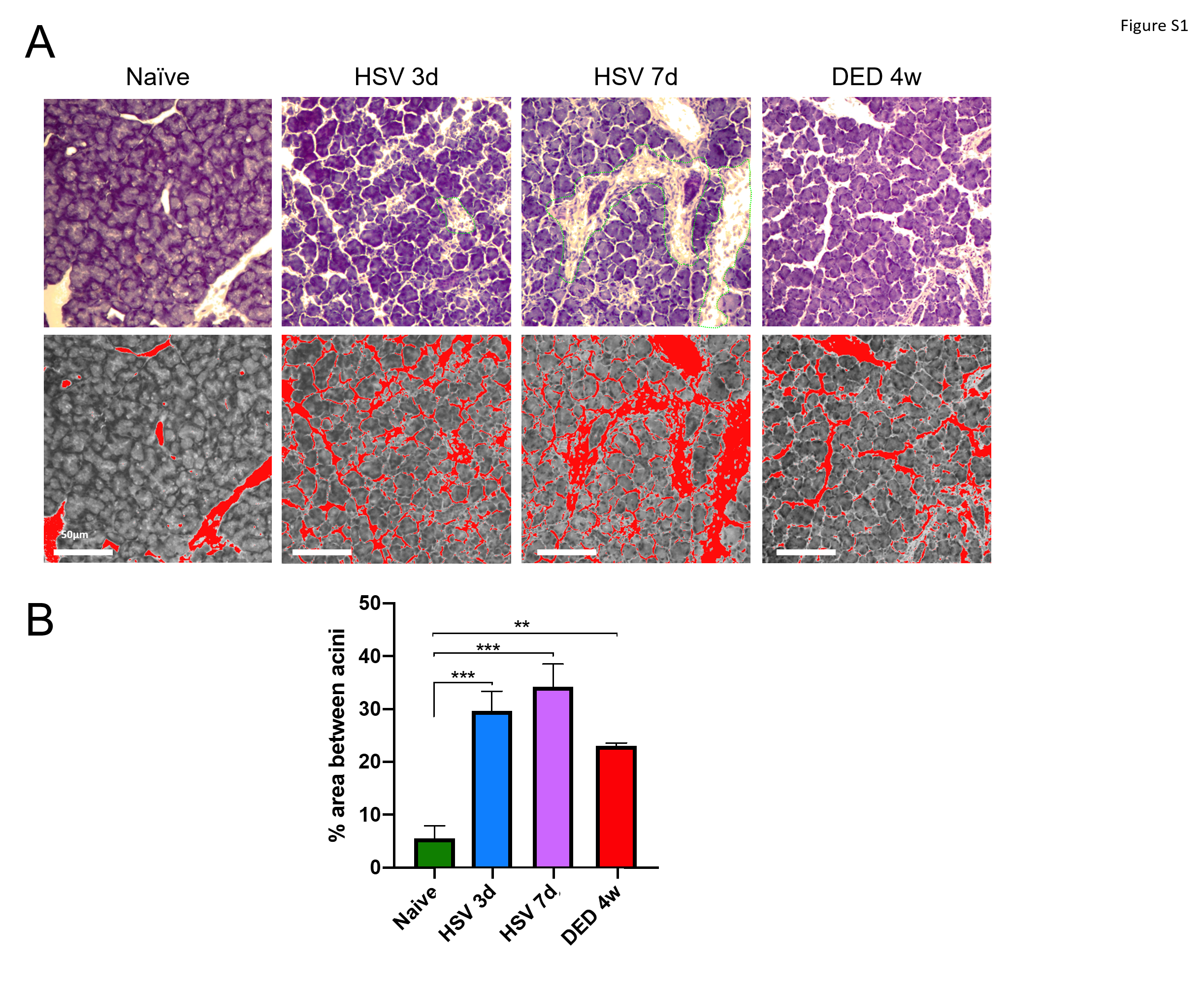

Supplement: Figure S1 — LG histopathology in naïve mice, 3 and 7 days post HSV-1 infection, and 4w post DED. (A) Converted images of LG histopathology using ImageJ software of all experimental groups. The red extent exhibits the area between acinar cells in all groups. (B) Quantification of the area between acinar cells expressed in percentage of area in red of total image area. Results are presented as mean ± SEM. One-way ANOVA and Bonferroni multiple comparison test. **p < 0.01, ***p < 0.001. [file Data_Sheet_1.zip › FigS1.tif]

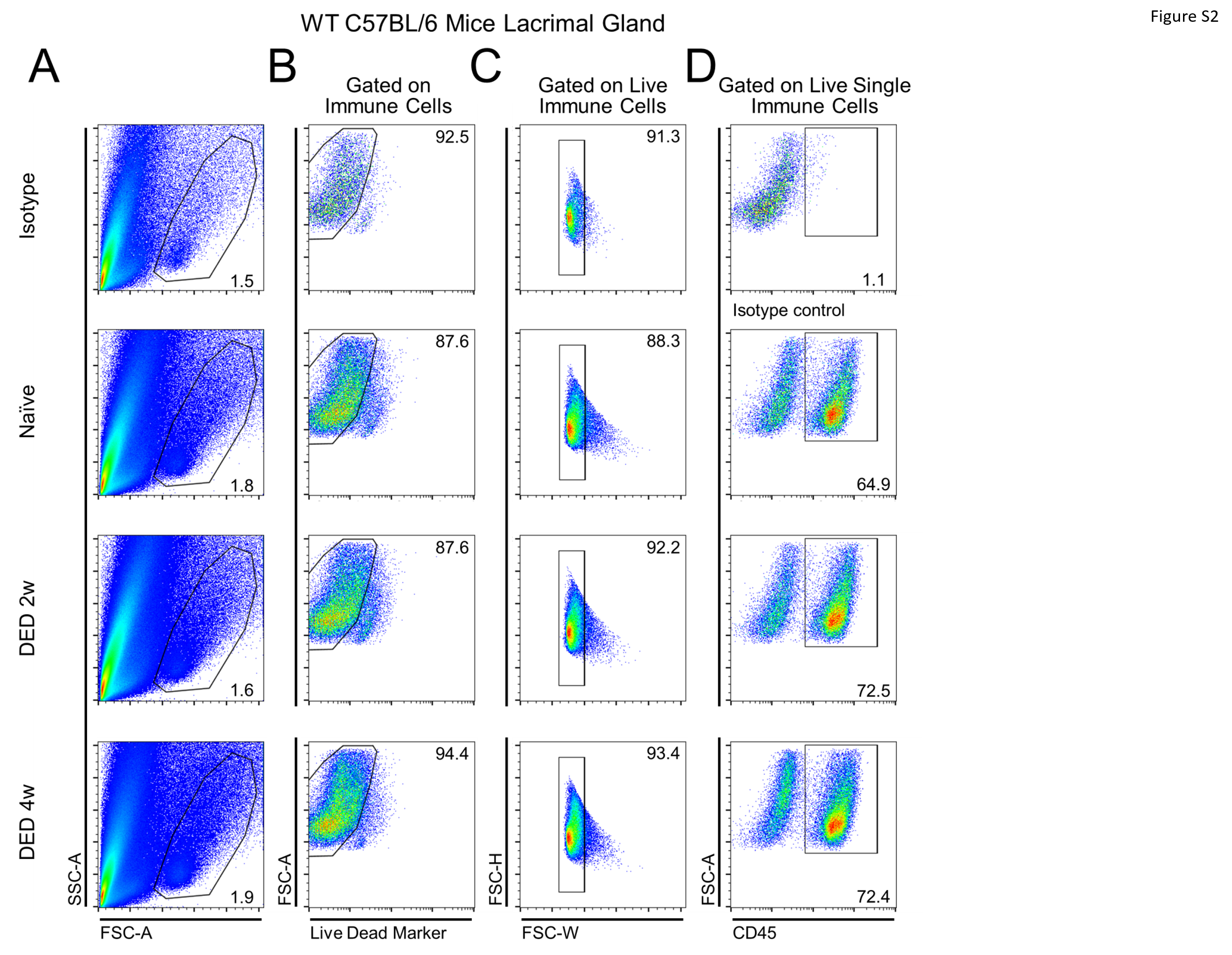

Supplement: Figure S1 — LG histopathology in naïve mice, 3 and 7 days post HSV-1 infection, and 4w post DED. (A) Converted images of LG histopathology using ImageJ software of all experimental groups. The red extent exhibits the area between acinar cells in all groups. (B) Quantification of the area between acinar cells expressed in percentage of area in red of total image area. Results are presented as mean ± SEM. One-way ANOVA and Bonferroni multiple comparison test. **p < 0.01, ***p < 0.001. [file Data_Sheet_1.zip › FigS2.tif]

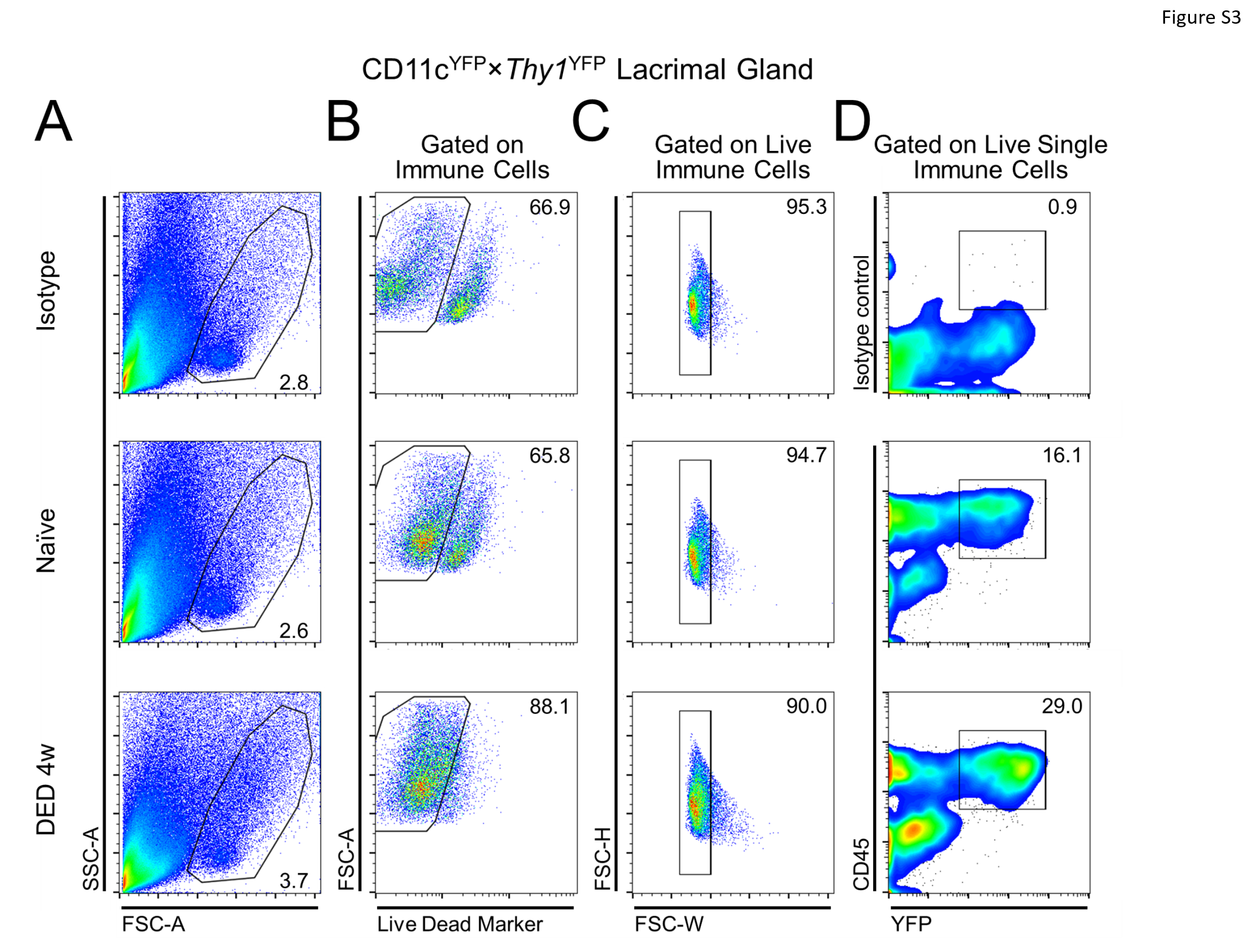

Supplement: Figure S1 — LG histopathology in naïve mice, 3 and 7 days post HSV-1 infection, and 4w post DED. (A) Converted images of LG histopathology using ImageJ software of all experimental groups. The red extent exhibits the area between acinar cells in all groups. (B) Quantification of the area between acinar cells expressed in percentage of area in red of total image area. Results are presented as mean ± SEM. One-way ANOVA and Bonferroni multiple comparison test. **p < 0.01, ***p < 0.001. [file Data_Sheet_1.zip › FigS3.tif]

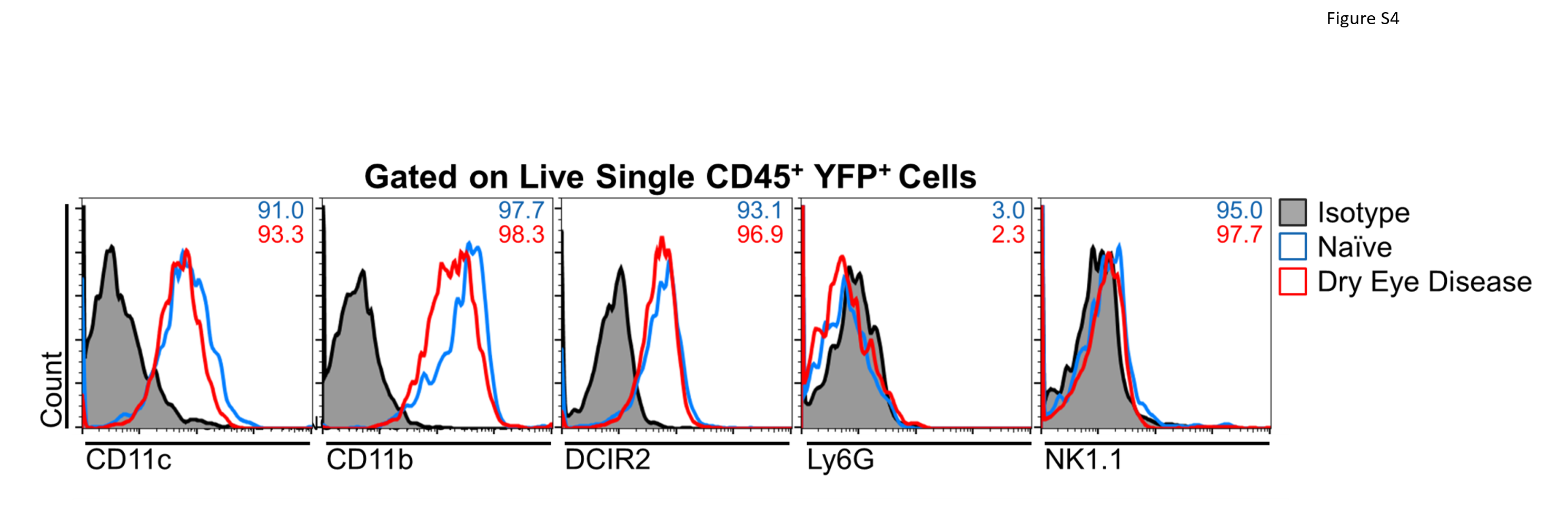

Supplement: Figure S1 — LG histopathology in naïve mice, 3 and 7 days post HSV-1 infection, and 4w post DED. (A) Converted images of LG histopathology using ImageJ software of all experimental groups. The red extent exhibits the area between acinar cells in all groups. (B) Quantification of the area between acinar cells expressed in percentage of area in red of total image area. Results are presented as mean ± SEM. One-way ANOVA and Bonferroni multiple comparison test. **p < 0.01, ***p < 0.001. [file Data_Sheet_1.zip › FigS4.tif]

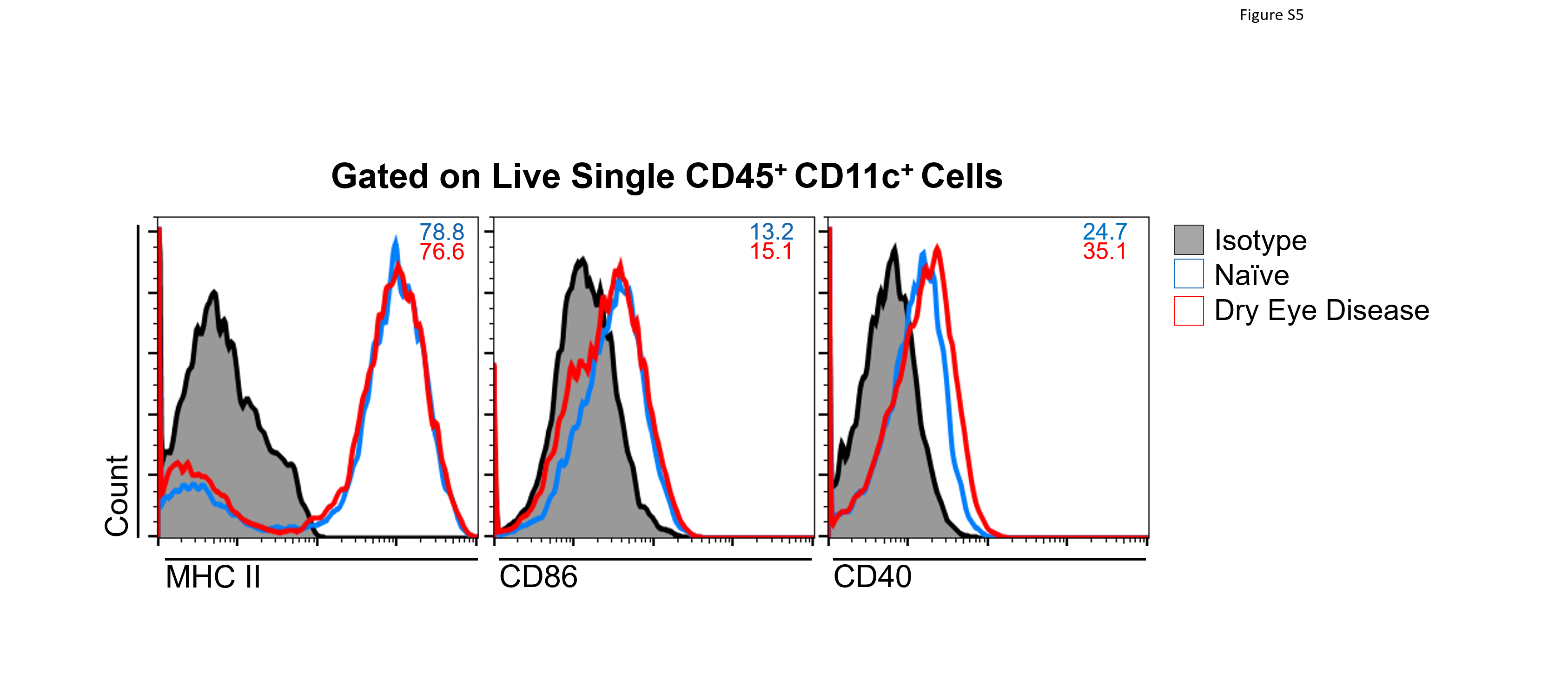

Supplement: Figure S1 — LG histopathology in naïve mice, 3 and 7 days post HSV-1 infection, and 4w post DED. (A) Converted images of LG histopathology using ImageJ software of all experimental groups. The red extent exhibits the area between acinar cells in all groups. (B) Quantification of the area between acinar cells expressed in percentage of area in red of total image area. Results are presented as mean ± SEM. One-way ANOVA and Bonferroni multiple comparison test. **p < 0.01, ***p < 0.001. [file Data_Sheet_1.zip › FigS5.tif]
