# Supplementary material for: Effect of Dry Eye Disease on the Kinetics of Lacrimal Gland Dendritic Cells as Visualized by Intravital Multi-Photon Microscopy
Source: Front Immunol. 2020 Aug 12;11:1713. doi: 10.3389/fimmu.2020.01713 (PMC7434984; doi:10.3389/fimmu.2020.01713)
Supplement: Table S1 — Antibody list. [file Table_1.pdf]

Supplementary Table S1: Antibody List

Marker	Clone	Vendor	Isotype
**CD45**	30-F11	Biolegend	Rat IgG2b, κ
**CD11b**	M1/70	BD Pharmingen	Rat IgG2b, κ
**CD11c**	HL3	BD Pharmingen	Hamster IgG1, λ2
**MHC II (I-A/I-E)**	M5/114.15.2	Biolegend	Rat IgG2b, κ
**CD40**	1C10	eBioscience	Rat IgG2a, κ
**CD86**	GL-1	Biolegend	Rat IgG2a, κ
**CD3**	17A2	BD Pharmingen	Rat IgG2b, κ
**CD4**	RM4-5	Biolegend	Rat IgG2a, κ
**CD44**	IM7	Biolegend	Rat IgG2b, κ
**CD62L**	MEL-14	Biolegend	Rat IgG2a, κ
**Fixable Live/Dead Blue**	---	ThermoFisher	---
**Viability e780**	---	eBioscience	---
**CellTrace Violet**	---	Invitrogen	---
